# Diesel exhaust particle exposure reduces expression of the epithelial tight junction protein Tricellulin

**DOI:** 10.1186/s12989-020-00383-x

**Published:** 2020-10-15

**Authors:** Timothy Smyth, Janelle Veazey, Sophia Eliseeva, David Chalupa, Alison Elder, Steve N. Georas

**Affiliations:** 1 Department of Environmental Medicine, University of Rochester, Rochester, NY USA; 2 Department of Microbiology and Immunology, University of Rochester, Rochester, NY USA; 3 Department of Medicine, Pulmonary and Critical Care, University of Rochester, Box 692, 601 Elmwood Ave, University of Rochester, Rochester, NY 14627 USA

**Keywords:** Diesel exhaust particles, Tricellulin, Tric, Airway epithelial barrier, Tight junction

## Abstract

**Background:**

While exposure to diesel exhaust particles has been linked to aberrant immune responses in allergic diseases such as asthma, little attention has been paid to their effects on the airway epithelial barrier. In this study, we sought to determine the effect of diesel exhaust exposure on airway epithelial barrier function and composition using in vitro and in vivo model systems.

**Methods:**

16HBE14o- human bronchial epithelial cells were grown on collagen coated Transwell inserts and exposed to 5 to 50 μg/cm^2^ SRM 2975 diesel particulate matter (DEP) suspended in cell culture medium or vehicle controls. Changes in barrier function were assessed by measuring transepithelial electrical resistance (TEER) and permeability to 4 kDa FITC Dextran. Neonatal BALB/c mice were exposed to aerosolized DEP (255 ± 89 μg/m^3^; 2 h per day for 5 days) and changes in the tight junction protein Tricellulin were assessed 2 weeks post exposure.

**Results:**

A six-hour incubation of epithelial cells with diesel exhaust particles caused a significant concentration-dependent reduction in epithelial barrier integrity as measured by decreased TEER and increased permeability to 4 kDa FITC-Dextran. This reduction in epithelial barrier integrity corresponded to a significant reduction in expression of the tight junction protein Tricellulin. siRNA mediated knockdown of Tricellulin recapitulated changes in barrier function caused by DEP exposure. Neonatal exposure to aerosolized DEP caused a significant reduction in lung Tricellulin 2 weeks post exposure at both the protein and mRNA level.

**Conclusion:**

Short term exposure to DEP causes a significant reduction in epithelial barrier integrity through a reduction in the tight junction protein Tricellulin. Neonatal exposure to aerosolized DEP caused a significant and sustained reduction in Tricellulin protein and mRNA in the lung, suggesting that early life exposure to inhaled DEP may cause lasting changes in airway epithelial barrier function.

## Background

Air pollution has long been linked to increased morbidity and mortality of pulmonary diseases such as COPD and asthma [1–4]. Increased exposure to airborne particulate matter (PM) has been shown to strongly correlate with negative health outcomes, including reduced lung function [5], increased hospitalizations [6], and premature death [7]. Diesel exhaust particles (DEP), which result from the incomplete combustion of diesel fuel, are a major component of airborne PM and have been largely studied for their role in enhancing immune responses in allergic diseases such as asthma. While multiple studies have documented the ability of inhaled DEP to impact and activate cells of the immune system [8–10], less is known about the effect of DEP on the airway epithelial barrier.

Airway epithelial cells form a physical barrier to inhaled allergens, particulates, and pathogens through a combination of secreted mucus and apical junctional complexes (AJCs) which form between neighboring cells [11]. The AJCs consist of the basolateral adherens junctions which are important for cell signaling and initiating cell-cell adhesion [12] while the apical tight junctions (TJs) are composed of the claudin family, tight junction-associated MARVEL proteins (TAMPs), and immunoglobulin-like proteins such as the junctional adhesion molecule family [13, 14]. Due to their apical location, TJs regulate paracellular passage of ions and macromolecules across the epithelium. Water and ion permeability are regulated primarily by the claudin protein family, while macromolecules are primarily regulated by the TAMP family [15]. The TAMP family shares the four-pass transmembrane MARVEL domain and is composed of Occludin, Tricellulin (Tric), and MarvelD3 [16].

Previous in vitro studies have shown that exposure to DEP causes epithelial barrier dysfunction as measured by reduced transepithelial electrical resistance (TEER) [17]. Similarly, DEP also causes barrier dysfunction in human aortic endothelial cells [18], although it is not clear how readily inhaled DEP would access the vasculature since only a small fraction of inhaled particles cross the pulmonary epithelium [19, 20]. In these reports, DEP did not significantly affect Occludin protein levels, suggesting that another tight junction protein may be affected by DEP exposure. Due to its location at tricellular contacts, which have been suggested to be a point of increased paracellular flux due to the formation of a transepithelial pore [21, 22], Tricellulin represents a possible target for DEP induced barrier dysfunction. As very little is known about the expression or function of Tricellulin in the airways, we sought to determine if exposure to DEP could cause a reduction in airway epithelial barrier function through a change in the MARVEL protein, Tricellulin.

## Results

### Diesel exhaust particles perturb the pulmonary epithelial barrier

**Fig. 1:**
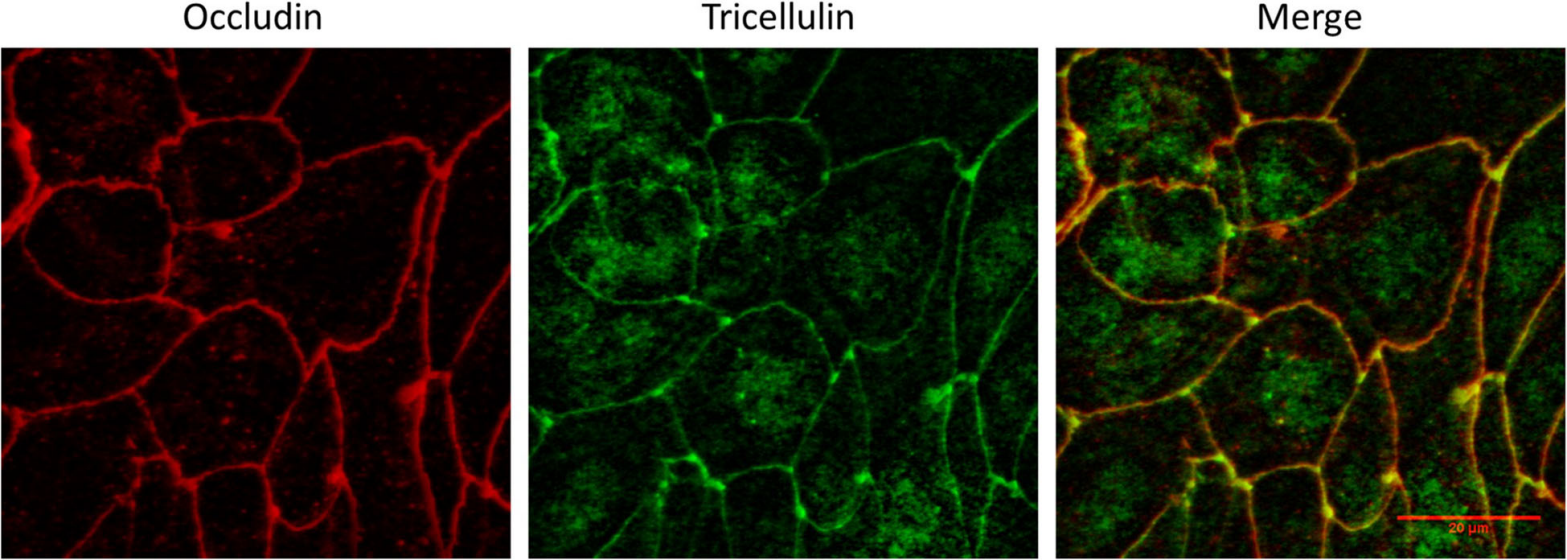
16HBE14o- Monolayers Exhibit Proper Localization of the Tight Junction Proteins Occludin and Tricellulin. Immunofluorescence staining of methanol fixed 16HBE14o- cells with anti-Occludin (red) and anti-Tricellulin (green). Tricellulin is predominantly visible at points of tricellular contact while Occludin is distributed along bicellular junctions. Scale bar 30 μm

When grown on collagen coated Transwell inserts, 16HBE14o- cells form complete monolayers that maintain differentiated epithelial morphology including formation of tight junctions [23]. In addition, 16HBE14o- monolayers display the expected localization of the tight junction proteins Occludin and Tricellulin (Fig. 1) as seen in other epithelial cell types [21, 24]. These factors allow for testing of the effects of various toxicants on epithelial barrier function. Changes in epithelial barrier function were assessed by measuring TEER and permeability of 4 kDa FITC-Dextran. TEER measures the ability of the epithelium to exclude ion flow across the epithelial sheet, with higher TEER representing a stronger barrier. In contrast, permeability to 4 kDa FITC-Dextran assesses the ability of the epithelium to regulate movement of macromolecules across the epithelial sheet, where lower permeability represents a stronger epithelial barrier.

**Fig. 2:**
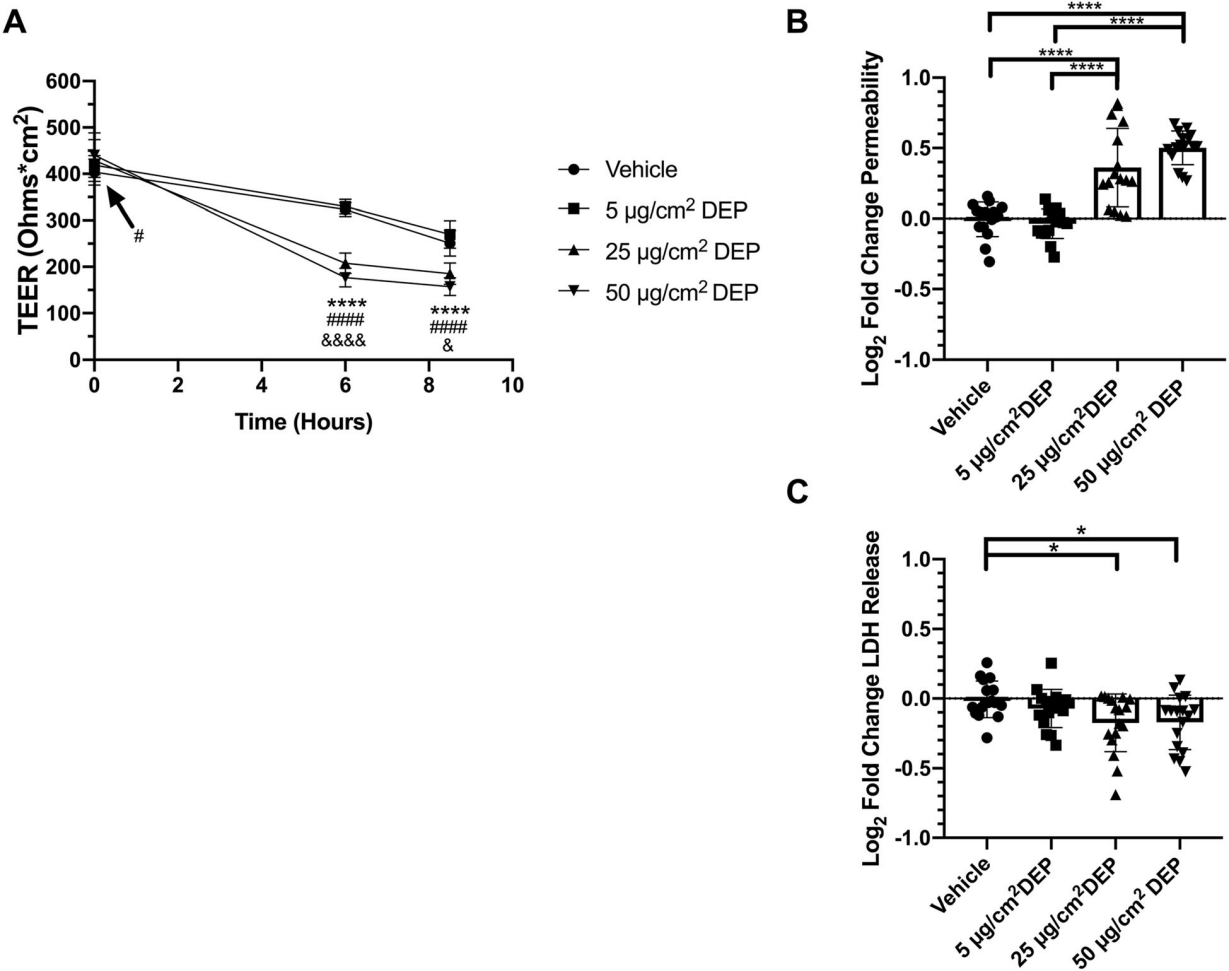
Short term exposure to DEP reduces epithelial barrier function without causing cytotoxicity. **a** TEER measurements of cells exposed to indicated concentrations of DEP at time 0, 6, and 8.5 h after application of DEP. One-Way ANOVA with Tukey’s HSD, ****P < 0.0001 Vehicle vs 25 μg/cm^2^ DEP, #*P* < 0.05, ####*P* < 0.0001 Vehicle vs 50 μg/cm^2^ DEP, &*P* < 0.05, &&&& < 0.0001 25 μg/cm^2^ vs 50 μg/cm^2^ DEP. Data from three independent experiments, *N* = 5–6 replicates per treatment per time point per experiment. **b** 4 kDa FITC-Dextran permeability measured at 8.5 h after application of DEP. One-Way ANOVA with Tukey’s HSD, ****P < 0.0001. Data from three independent experiments, *N* = 5–6 replicates per treatment per experiment. **c** Fold change LDH release normalized to average Vehicle LDH release. One-Way ANOVA, *P < 0.05. Data from three independent experiments, *N* = 5–6 replicates per treatment per time point per experiment

In order to test the effects of DEP on the pulmonary epithelial barrier, 16HBE14o- model monolayers were exposed to varying concentrations of DEP suspended in cell culture medium or Vehicle controls for 6 h. Using the in vitro Sedimentation, Diffusion and Dosimetry model (ISDD) [25] with reported values for media viscosity and density [25], as well as the raw and effective density [26, 27] and the diameter of the diesel particles/agglomerates (see Methods), we estimate that 100% of the applied dose deposited onto the cell surface within the first 2 h of the six-hour exposure window (Data not shown). Following these six-hour exposures to DEP, TEER was significantly reduced in both 25 and 50 μg/cm^2^ DEP exposed wells, but not 5 μg/cm^2^ exposed wells, when compared to Vehicle controls (Fig. 2a). In addition, TEER was significantly higher in wells exposed to 5 μg/cm^2^ DEP compared to those exposed to either 25 or 50 μg/cm^2^, and wells exposed to 25 μg/cm^2^ DEP were found to be significantly higher than those exposed to 50 μg/cm^2^. We measured TEER again at the conclusion of the permeablity assay, and noticed a slight reduction in the rate of decline in TEER between 6 and 8.5 h after DEP application in cells exposed 25 or 50 μg/cm^2^, compared to Vehicle control wells or cells exposed to 5 μg/cm^2^ DEP (Fig. 2a). Since medium was aspirated from the wells before FITC-dextran solution was applied, this could reflect a reduction in the concentration of DEP remaining during the FITC-dextran assay. The changes in TEER were further visualized as percent of Vehicle TEER, indicating that exposure to 25 or 50 μg/cm^2^ DEP induced approximately a 35 or 45% reduction of TEER by 6 h after exposure, respectively (Supplemental Figure 1). These findings suggested DEP reduces epithelial electrical resistance in 16HBE14o- monolayers in a concentration-dependent manner.

In conjunction with TEER measurements, we assessed the ability of 16HBE14o- monolayers to exclude macromolecule passage across the epithelial sheet using a FITC-Dextran permeability assay. FITC-Dextran was added to the apical surface of the cells 6 h after beginning exposures, and samples from the basolateral compartment were measured 2.5 h later. Similar to TEER measurements, cells exposed to either 25 or 50 μg/cm^2^ DEP exhibited reduced barrier function as shown by significantly increased permeability to 4 kDa FITC-Dextran when compared to Vehicle treated cells. As with the TEER measurements, 5 μg/cm^2^ DEP exposed cells showed no change in barrier function compared to Vehicle controls (Fig. 2b). No difference in permeability was observed between cells exposed to 25 or 50 μg/cm^2^ DEP. The defects observed in the epithelial barrier occurred in the absence of any detectable cytotoxicity as measured by an LDH release assay (Fig. 2c), suggesting that DEP reduces barrier function independent of cell death.

### The tight junction protein Tricellulin is reduced by DEP exposure

**Fig. 3:**
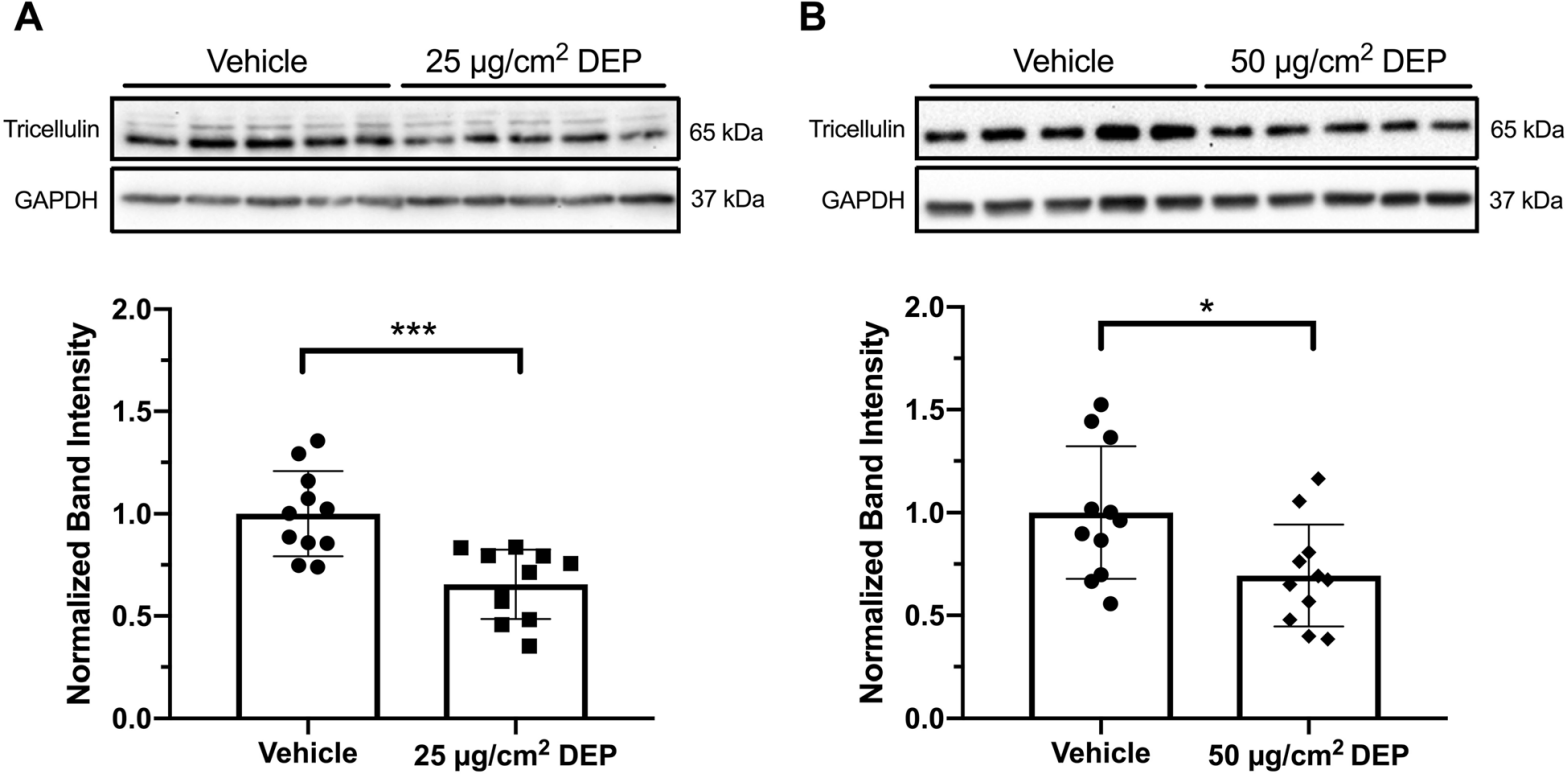
Six-hour exposure to DEP causes a reduction in Tricellulin protein. **a** Cells exposed to 25 μg/cm^2^ DEP or **b** 50 μg/cm^2^ DEP for 6 h demonstrate a reduction in Tricellulin protein as measured by whole cell lysate Western blot. Representative Western blot from one experiment, densitometry results from two independent experiments. Student’s t-test, *P < 0.05, ***P < 0.001, *N* = 5–6 replicates per treatment per experiment

Due to its concentration at points of tricellular contacts, which are potential sites of increased macromolecule flux, we measured Tricellulin protein in cells exposed to DEP. In order to determine if DEP affected Tricellulin expression, we measured its expression in 16HBE14o- cells exposed to 25 and 50 μg/cm^2^ DEP, which caused similar changes in macromolecule permeability (Fig. 2b). We found a significant reduction in Tricellulin protein in whole cell lysates of both 25 and 50 μg/cm^2^ DEP exposed cells (Fig. 3), averaging 65 ± 17 and 69 ± 25% reduction compared to vehicle treated cells, respectively. Results using 50 μg/cm^2^ DEP were more variable, but still significantly different compared to control wells on average. These results indicate that Tricellulin may be particularly vulnerable to pollutant insults such as DEP and may drive barrier dysfunction following exposure.

### Tricellulin knockdown recapitulates the effects of DEP exposure

As Tricellulin was found to be specifically reduced following DEP exposure, we wanted to understand whether Tricellulin had a role in maintaining airway epithelial barrier function. Tricellulin has been shown to regulate barrier function in other tissues [21, 24], but whether it contributes to airway epithelial barrier integrity has not been previously reported (to our knowledge). To explore the role of Tricellulin, sub-confluent epithelial monolayers were transfected with siRNA targeted against Tricellulin or scramble controls and grown on collagen coated Transwells. In order to determine if Tricellulin knockdown would fully recapitulate changes in the epithelial barrier caused by DEP exposure, we measured both changes in TEER after knockdown and permeability to 4 kDa FITC-Dextran.

**Fig. 4:**
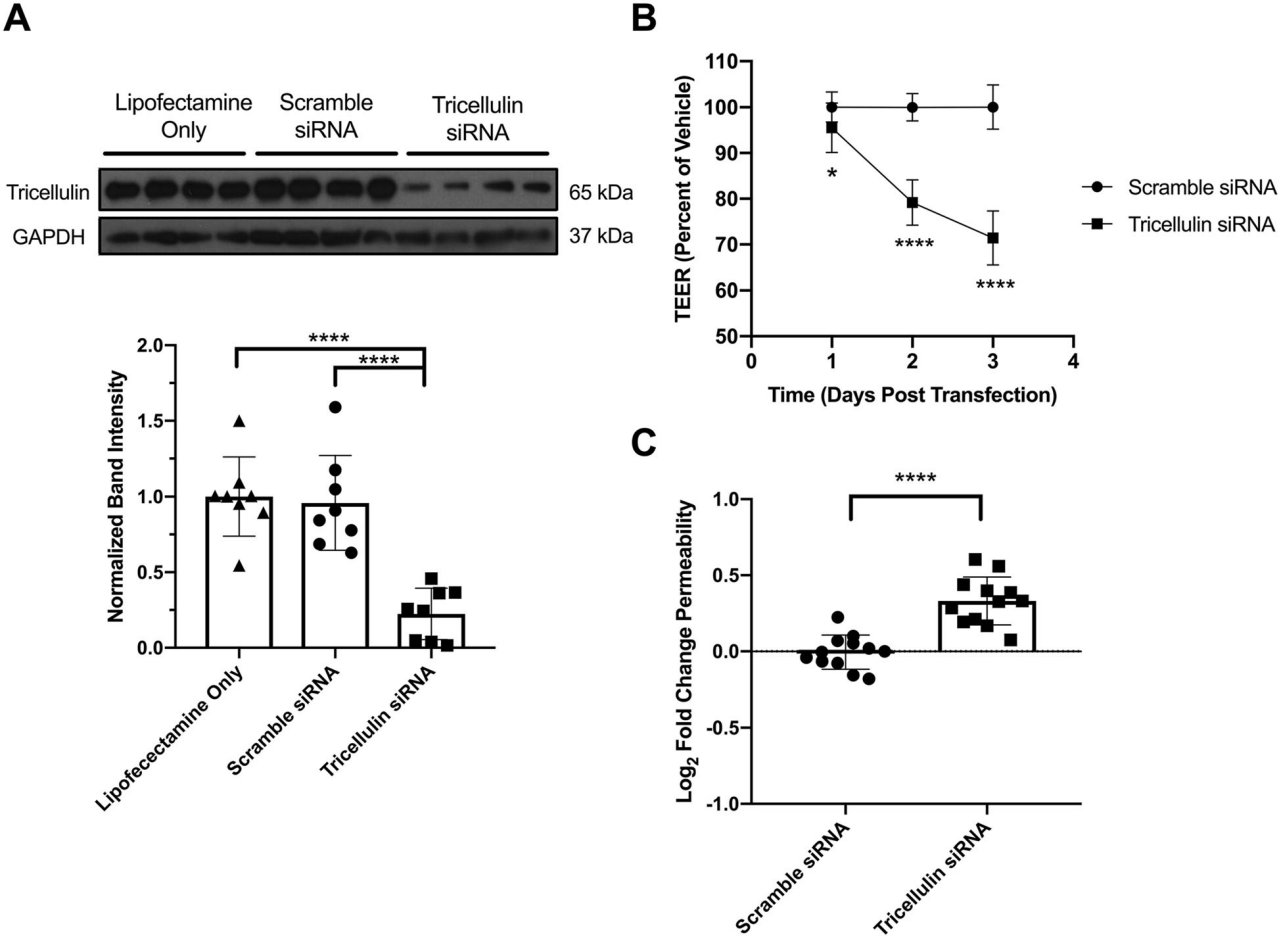
siRNA mediated knockdown of Tricellulin recapitulates barrier dysfunction caused by DEP exposure. **a** Tricellulin siRNA significantly reduces Tricellulin protein 3 days post transfection. One-Way ANOVA with Tukey’s HSD, **P < 0.01. Representative Western blot from one experiment, densitometry results from two independent experiments, *N* = 4 replicates per treatment per experiment. **b** Tricellulin knockdown causes a reduction in TEER. Student’s t-test with Holm-Sidak method, *P < 0.05, ****P < 0.0001. Data from three independent experiments, *N* = 4 replicates per experiment per time point. **c** Tricellulin knockdown cells exhibit increased permeability to 4 kDa FITC-Dextran 3 days post transfection. Student’s t-test, ****P < 0.0001. Data from three independent experiments, *N* = 4 replicates per treatment per experiment

Three days post transfection, Tricellulin protein levels were significantly reduced compared to lipofectamine controls, as well as scramble siRNA controls (Fig. 4a). This reduction in protein corresponded to a significant decrease in TEER compared to scramble transfected cells (Fig. 4b), as well as a significant increase in permeability to 4 kDa FITC-Dextran (Fig. 4c). These results establish a role for Tricellulin in maintaining airway epithelial barrier integrity, and also show that a partial reduction in Tricellulin protein is sufficient to recapitulate the DEP-mediated barrier disruption.

### Early life exposure to inhaled DEP causes a lasting reduction in lung Tricellulin expression

To test the impact of DEP on Tricellulin expression in the lung, neonatal BALB/c mice (PND 3–5) were exposed by whole body inhalation to aerosolized DEP for 2 h per day for five consecutive days. We used a neonatal exposure model because of the substantial evidence indicating that early life exposures represent a particularly vulnerable period for adverse effects of inhaled pollutants [28–32]. The aerosolized DEPs averaged 193 nm in thermodynamic diameter with a CMD of 1.8 (see Methods). These aerosols were further characterized as containing 2.2 to 2.6 × 10^4^ particles/cm^3^ with a mean particle count concentration of 2.4 × 10^4^ particles/cm^3^. The aerosol mass concentration ranged from 197 to 322 μg DEP/m^3^ with a mean concentration of 255 ± 89 μg/m^3^ over the 5 days of exposure. Using the Multi-Path Particle Dosimetry Model [33] (MPPD v 3.04) and allometric/surface area scaling, as described in Methods, we calculated respiratory tract deposition fractions of 4.3% in the tracheobronchial region and 7.3% in the alveolar region. Using these modelled deposition fractions, we calculated a deposited mass dose of 0.64 μg in adult BALB/c mouse lower respiratory tract. Scaling this value to the surface area of PND 5 mouse lungs [34], we estimated a deposited dose of 12.7 ng/cm^2^ over the total 10 h of aerosol exposure.

**Fig. 5:**
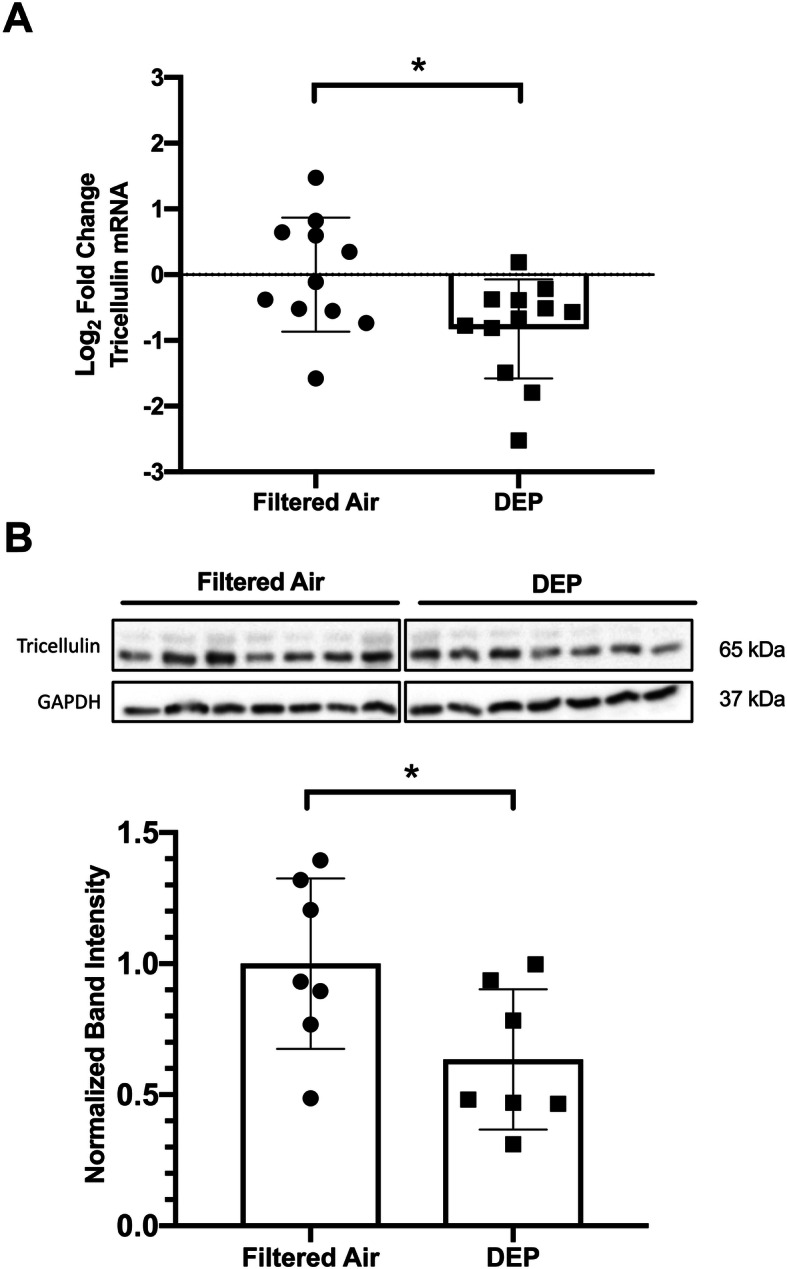
Early life exposure to DEP causes a reduction in Tricellulin expression in the lung. **a** Neonatal mice exposed to aerosolized DEP exhibit a reduction in Tricellulin mRNA in the lung 2 weeks post final exposure as measured by RT-qPCR. Unequal variances t-test, *P < 0.05, *N* = 11 (FA) or *N* = 12 (DEP). **b** Neonatal mice exposed to aerosolized DEP exhibit a reduction in Tricellulin protein in the lung 2 weeks post final exposure as measured by Western blot. Student’s t-test, *P < 0.05, *N* = 7 per treatment

Two weeks after the final exposure, mice were sacrificed, and protein and mRNA were isolated from whole lungs. Following these exposures, Tricellulin mRNA was found to be significantly reduced in DEP exposed lungs when compared to filtered air (FA) exposed lungs (Fig. 5a). Importantly, this reduction in mRNA corresponded to a significant reduction in Tricellulin protein in whole lungs (Fig. 5b). Similar changes in Tricellulin protein were observed in a separate cohort of neonatal BALB/c mice similarly exposed by whole body inhalation starting on PND 4–7 (Supplement 2). As these changes were measurable 2 weeks after the final exposure, DEP may cause a lasting reduction in Tricellulin expression in the lung in early life, possibly leading to a lasting reduction in epithelial barrier integrity.

## Discussion

Exposure to airborne PM has long been linked to increased pulmonary disease morbidity and mortality [1–4]. Exposure to traffic-related air pollution has also been linked to the development of pulmonary conditions such as asthma [5, 35–37]. While PM like DEP has been shown to act as an inhaled adjuvant, enhancing the immune response to common allergens such as house dust mite [38] and ragweed pollen [39], little work has investigated the impact of DEP on the epithelial barrier function of the lung. The present study demonstrates that exposure to DEP can cause a reduction in epithelial barrier function through a reduction in the tight junction protein Tricellulin.

Tricellulin, also known as Tric, is a member of the tight junction–associated MARVEL protein (TAMP) family. Along with the other members of this family – Occludin and MarvelD3 – it has a tetra-spanning MARVEL (MAL and related proteins for vesicle trafficking and membrane link) domain [16]. First discovered in 2005, Tricellulin was shown to localize primarily to points of tricellular contact, where it extends apically to basolaterally down the epithelial sheet [21]. Despite the shared MARVEL domain, Tricellulin has been shown to lack interactions with Occludin, instead forming strong homotypic interactions due to structural differences between the extracellular loop 2 (ECL2) of each protein [16, 40]. The structure of Tricellulin’s ECL2 allows for interaction between Tricellulin molecules of three adjacent cells at tricellular contacts, rather than the interactions between two adjacent cells at bicellular contacts caused by Occludin.

These tricellular contacts are thought to be a natural weak point in the epithelial sheet, creating a “central pore” of approximately 10 nm that has been observed by freeze fracture microscopy [41]. These pores have been suggested as sites of increased paracellular permeability, as they represent a gap in the barrier formed by cell-cell contact [21, 22]. A loss of Tricellulin has been shown to compromise barrier function as measured by reduced TEER and increased permeability to 4 kDa FITC-Dextran in EpH4 mammary epithelial cells [21], while overexpression of Tricellulin has shown to increase barrier function, reducing permeability of macromolecules in a size dependent manner in Madin-Darby canine kidney cells [24]. Despite these studies in various epithelial cell types, little is known about the function of Tricellulin in airway epithelial barrier function. Due to its role in sealing tricellular junctions, loss of Tricellulin may cause an unexpectedly high reduction in the airway epithelium’s ability to regulate movement of macromolecules across the epithelium.

We found that exposure to DEP caused a reduction in Tricellulin protein expression by 6 h after exposure (Fig. 3). Importantly, the concentrations of DEP used in these experiments are comparable to those used in recent publications [17, 42, 43] and did not induce any detectable levels of cytotoxicity as measured by LDH release (Fig. 2c). While measurments of LDH release has been shown to be impacted by the presence of particulate matter such as TiO_2_ and flame soot [44, 45] due to interactions between the particles and LDH, diesel exhaust particles have been shown to have minimal impact on LDH activity [44]. This effect, accounting for approximately 10% less LDH activity due to the presence of DEP, may account for the slight reduction in measured LDH release observed in 25 and 50 μg/cm^2^ DEP exposed wells (Fig. 2c). This finding further supports that diesel exhaust particles are not cytotoxic to 16HBE14o- cells at concentrations used in our studies.

Previous studies in which 16HBE14o- cells were exposed to SRM 2975 showed no change in Occludin mRNA following 24 h exposure despite a significant reduction in TEER [17]. In addition, primary rat airway epithelial cells (AECs) and human A549 cells showed no change in Occludin protein by whole cell lysate following 3 h exposure to 20 μg/cm^2^ DEP [42]. DEPs inability to affect Occludin protein levels was further supported in a model of endothelial DEP exposure, where Occludin protein was found to be unchanged following six-hour exposures to two different types of DEP in human aortic endothelial cells [18]. As previous studies have shown that DEP does not affect Occludin protein levels, a reduction in Tricellulin protein may explain changes in barrier function observed following DEP exposure.

While Occludin has not been shown to be reduced at the mRNA or protein level following DEP exposure, these studies have noted changes in its localization to the plasma membrane. Twenty four hours exposure to SRM 2975 causes increased cytoplasmic staining for Occludin in 16HBE14o- cells [17], while primary rat AECs and human A549 cells exposed to 20 μg/cm^2^ DEP for 3 h exhibited a reduction of Occludin present at the plasma membrane despite no change in Occludin protein levels in whole cell lysates [42]. Despite this possibility of Occludin reorganization following DEP exposure, our study demonstrated that a loss of Tricellulin through siRNA mediated knockdown can cause a significant decrease in barrier function as measured by both reduced TEER and increased permeability to FITC-Dextran, suggesting that a loss of Tricellulin is sufficient to significantly impact barrier function. Exposure to traffic related air pollution such as diesel exhaust has been implicated in the development and exacerbation of pulmonary diseases including asthma [5, 35–37]. This relation has mostly been linked to the adjuvanticity of PM [46–48], with little attention paid to changes in the pulmonary epithelium due to such exposures. Our experiments show that exposure to traffic related air pollution can directly compromise epithelial barrier function. There are several potential consequences of epithelial barrier dysfunction in asthma. First, subjects with leaky airways might be more susceptible to airway inflammation caused by inhaled particles and allergens. Second, barrier dysfunction might pre-dispose to respiratory viral infections, which are a known cause of asthma exacerbations. Third, barrier dysfunction is known to activate intracellular signaling cascades in epithelial cells, leading to cell activation and differentiation. Emerging work has shown that airway epithelial barrier dysfunction is a common feature in asthma [11]. For example, immunohistochemical staining performed on bronchial biopsy samples from asthmatic lungs have shown reduced levels of ZO-1 [49], α-catenin, and E-cadherin [50] when compared to non-asthmatic lungs. In addition, epithelial cells derived from cadaveric lungs of asthmatics have shown stable reductions in E-cadherin [51] and β-catenin [52] staining compared to non-asthmatics when propagated at air-liquid interface, suggesting that the asthmatic lung epithelium develops durable changes in junctional protein composition.

While changes in epithelial barrier function may be a consequence of asthma and airway inflammation, these observations raise the possibility that perturbations in the pulmonary epithelial barrier may in fact occur due to environmental exposures during development. Interestingly, our neonatal exposure model (Fig. 5) shows that early life exposure to inhaled DEP causes a reduction in Tricellulin protein and mRNA in the lung that persists through 2 weeks after exposure, suggesting a durable change in junctional protein composition. The idea that early life exposures to inhaled pollutants can cause lasting changes in lung structure are not without precedence, since mice exposed to inhaled vehicle derived PM_2.5_ from embryonic day 5.5 to PND 39 showed reduced alveolar number and increased alveolar spaces at PND 40 [53]. In addition, rats exposed to combustion generated ultrafine particles from PND 7–25 were shown to have changes in lung structure and mechanics at 81 days of age [54], while PND 10 rats exposed to aerosolized soot and iron particles for 3 days displayed reduced cell proliferation in the proximal alveolar region of the lung [55]. These studies demonstrate that early life exposure to particulate matter can cause significant and sustained changes in the structure of the lung. While future studies are required to determine the persistence of the DEP induced reduction in lung Tricellulin over the lifespan and its consequences for barrier function, our work raises the possibility that neonatal exposure to DEP may stably reduce epithelial barrier function in part through a reduction in the tight junction protein, Tricellulin.

We acknowledge that we do not report the precise mechanism by which DEP inhibits Tricellulin expression but speculate generation of reactive oxygen species (ROS) may be involved. Numerous studies have shown increased ROS generation following exposure to DEP [56–58]. This ROS generation has been implicated in many downstream pathways, including the activation of mitogen activated protein kinases (MAPKs) such as extracellular signal-regulated protein kinases (ERKs) [56] and c-Jun N-terminal Kinase (JNK) [57]. In addition, DEP can activate the downstream transcription factor AP-1 [59], which, along with JNK, have been shown to affect Tricellulin expression and localization in other epithelial tissues [60–62]. Future studies will be needed to determine the precise pathways through which DEP induced ROS generation may cause changes in Tricellulin expression.

We used both in vitro and in vivo model systems to study the effect of DEP exposure on Tricellulin expression. Although we observed that DEP exposure inhibited Tricellulin expression both in vitro and in vivo, there are clearly many differences between the two model systems that should be considered. First, our use of a transformed cell line does not represent a direct analog to the entirety of airway epithelial cells seen in the developing mouse lung. While 16HBE14o- monolayers have been shown to exhibit classic “cobblestone” patterning and cytokeratin filament organization seen in epithelial cells [23], they are nonetheless isolated solely from the bronchial surface and propagated at liquid-liquid interface on collagen coated polyester membranes, and cannot be used to fully interrogate the effects of DEP exposure on all segments of the developing neonatal lung.

Secondly, we do not assert that the particle dosimetry is equivalent in the in vitro and in vivo models. Using Using the Multi-Path Particle Dosimetry Model v 3.04 (MPPD v 3.04) [33], we estimated a total respiratory tract deposited dose of 2.4 μg in the ***adult mouse***. In order to account for differences in the adult and neonatal lung, we calculated the deposited dose over the entire surface area of the respiratory tract (adult mouse lung, ~ 500 cm^2^; PND 5 mouse lung, ~ 50 cm^2^ [34];). This yields a value of 48 ng/cm^2^ that was predicted to be deposited in the total respiratory tract, with an estimated deposited dose of 12.7 ng/cm^2^ specifically within the lower respiratory tract. Using the in vitro Sedimentation, Diffusion and Dosimetry model (ISDD) [25] with reported values for media viscosity and density [25], as well as the raw and effective density [26, 27] and the diameter of the diesel particles/agglomerates, we estimated 100% deposition of the applied diesel particles within the six-hour exposure window, corresponding to 8.25 to 16.5 μg deposited in 25 and 50 μg/cm^2^ DEP exposed wells, respectively. Therefore, this predicted in vivo surface area dose for a neonate is far lower than what was applied in the in vitro model.

Lastly, unlike in an intact lung, cell culture models lack a mechanism for clearing particles. In the intact lung, the combined activity of mucociliary function and, to a lesser degree, macrophage phagocytosis, would serve to remove particles from the airways. However, previous studies have shown cilia generation occurs steadily in mice until PND 21 in the trachea, lobar bronchi, and terminal bronchi [63] with cilia generated flow gradually increases in the trachea until plateauing at PND 9 [64]. These findings suggest neonatal mice may retain inhaled PM in the lung longer than similarly exposed adults, further reinforcing early life as a period of increased vulnerability to air pollution. Despite these clear differences between our in vitro and in vivo models, it is intriguing that both model systems supported similar mechanistic conclusions about the role of Tricellulin.

## Conclusions

In summary, this study demonstrates for the first time that exposure to DEP causes a significant reduction in epithelial barrier function as measured by reduced TEER and increased permeability to 4 kDa FITC-Dextran associated with a reduction in the tight junction protein Tricellulin. We further show that siRNA-mediated knockdown of Tricellulin is able to recapitulate the changes in barrier function observed following DEP exposure, suggesting Tricellulin itself is necessary for maintaining normal epithelial barrier function. Finally, this study demonstrates that early life exposure to inhaled DEP causes a reduction in Tricellulin expression at both the mRNA and protein levels 14 days post exposure, suggesting that early life exposure to DEP may cause a lasting change in lung structure and function later in life.

## Methods

### Cell culture

16HBE14o- human bronchial epithelial cells (a gift from Dr. D. C. Gruenert, University of California) were cultured in 16HBE14o- medium (high glucose Dulbecco’s Minimum Essential Medium with sodium pyruvate and L-glutamine (Life Technologies; Carlsbad, CA), 100 U/ml Penicillin and 100 μg/mL Streptomycin (Life Technologies), and 10 mmol/L HEPES (Life Technologies)) supplemented with 10% fetal bovine serum (Tissue Culture Biologicals; Tulane, CA). For diesel exhaust particle exposures, 1.5 × 10^5^ 16HBE14o- cells were seeded onto collagen coated, permeable polyester Transwell inserts with 0.4 μm pores and 0.33 cm^2^ growth area (Corning; Kennebunk, ME) and cultured in an atmosphere of 95% air/5% CO_2_ at 37 °C for 5 days. Serum concentration was reduced to 5% starting 18 h prior to treatment with DEP in order to transition cells to lower serum conditions prior to DEP exposure.

### Immunofluorescence

1.5 × 10^5^ 16HBE14o- cells were seeded onto collagen coated coverslips (18 mm diameter, thickness #1, Fisherbrand; Waltham, MA) and cultured in 16HBE14o- medium supplemented with 10% fetal bovine serum in an atmosphere of 95% air/5% CO_2_ at 37 °C for 6 days. Cells were fixed using methanol and stained as follows. Briefly, coverslips were fixed in ice cold 100% methanol (Fisher Chemical; Waltham, MA) for 15 min at − 20 °C. Fixed coverslips were blocked with 1x Dulbecco’s Phosphate Buffered Saline (DPBS, Life Technologies) + 5% normal donkey serum (Jackson ImmunoResearch Laboratories; West Groves, PA) + 0.3% Triton X-100 (Fisher Bioreagents; Waltham, MA) for 1 h at room temperature and incubated with 2 μg/mL rabbit anti-Tricellulin (Invitrogen 48–8400) and mouse anti-Occludin (Invitrogen 33–1500) in antibody dilution buffer (1x DPBS + 1% Bovine serum albumin (BSA, Sigma-Aldrich; St. Louis, MO) + 0.3% Triton X-100) overnight at 4 °C. Coverslips were then incubated with 4 μg/mL Alexa Fluor 568 donkey anti-mouse (Invitrogen A10037) and Alexa Fluor 488 donkey anti-rabbit (Invitrogen A21206) in antibody dilution buffer for 1 h at room temperature protected from light, followed by an incubation with 300 nM DAPI (Invitrogen) for 5 min protected from light. Coverslips were washed three times with 1x DPBS for 5 min per wash between each step. Coverslips were blotted dry, and mounted with 10 μL ProLong Gold anti-fade reagent (Invitrogen). Coverslips were cured for 24 h at room temperature protected from light and imaged with an Olympus FV1000 laser scanning confocal microscope (Olympus; Tokyo, Japan) at 60x magnification. Images were assembled using ImageJ software.

### Diesel exhaust particle exposure

Standard reference material 2975 (SRM 2975) was purchased from the National Institutes of Standards and Technology (Gaithersburg, MD). SRM 2975 was suspended to a concentration of 10 mg/mL in DPBS (Life Technologies) containing 0.05% Tween-20 (Bio-Rad; Hercules, CA). The resulting suspension was either sonicated with a Branson Sonifier 450 with cup horn attachment (Branson Ultrasonics; Danbury, CT) for 5 s on/off for a total time of 10 min, power setting 9, immediately before application, or with a Branson Sonifier 150 probe sonicator for 10 s on/off for a total time of 30 s, power setting 5, before being frozen at − 80 °C. Immediately prior to application, DEP was thawed, vortexed for 10 s, and sonicated with a Aquasonic 75 T water bath sonicator (VWR International; Radnor PA) for 15 min at 4 °C. DEPs were added to 16HBE14o- medium containing 1% fetal bovine serum to concentrations of 16.5, 82.5, or 165 μg/mL. One hundred microliters of resulting solutions were added to apical Transwell chambers, resulting in final applied concentrations of 5, 25, or 50 μg/cm^2^. 1% fetal bovine serum 16HBE14o- medium was added to the basolateral chamber at the time of treatment. The time of addition of DEP was defined as time zero (T0).

### In vitro dosimetry estimations

Estimations of the fraction of applied particles deposited onto cells were made using the in vitro Sedimentation, Diffusion and Dosimetry model (ISDD) [25]. Values for media viscosity (0.0006913 Pa-s) and density (1.007 g/cm^3^) at 37 °C were estimated according to values of similar cell culture media reported in [25]. The raw material density (1.27 g/cm^3^) and agglomerate effective density (1.78 g/cm^3^) of diesel exhaust particles were assigned according to values reported in [26, 27]. The particle/agglomerate diameters were measured to be 1100 nm by dynamic light scattering using a Malvern Zetasizer Nano ZS (Malvern Instruments; UK) with the following specifications: temperature, 25 °C; material refractive index, 1.59; material absorption, 0.01; dispersant refractive index, 1.33; dispersant viscosity, 0.8881 cP. Media column height was defined as 3 mm, and a total simulation time was defined as 6 h.

### Transepithelial electrical resistance

TEER measurements were performed with an EVOMX volt-ohm-meter (World Precision Instruments; Sarasota, FL) on 16HBE14o- human bronchial epithelial cells grown on collagen coated, permeable polyester Transwell inserts with 0.4 μm pores and 0.33 cm^2^ growth area (Corning). TEER was measured at different time points after addition of DEP, as indicated in Figure Legends.

### FITC-Dextran permeability assay

Paracellular permeability of fluorescent macromolecules was investigated by measuring passage of apically applied 4 kDa FITC-Dextran (Sigma-Aldrich) across epithelial monolayers. FITC-Dextran was suspended to a concentration of 10 mg/mL in phenol-free high glucose Dulbecco’s Minimum Essential Medium with L-glutamine and HEPES (Life Technologies), added to the apical chamber, and samples were collected from the basolateral chamber 2.5 h later. Sample fluorescence was measured using a Beckman Coulter DTX 880 multimode detector and the concentration of FITC-Dextran in the basolateral chamber was calculated using a standard curve.

### Cytotoxicity assay

The CytoTox 96 Non-Radioactive Cytotoxicity Assay (Promega; Madison, WI) was performed according to the manufacturer’s instructions on 16HBE14o- human bronchial epithelial cells grown on collagen coated Transwell inserts (Corning) following six-hour exposure to 5 to 50 μg/cm^2^ DEP or Vehicle controls. Briefly, at the time of assessment, apical well media was collected and diluted 1:25 in DPBS (Life Technologies) and 50 μl of the resulting medium was combined with 50 μl CytoTox 96 reagent and incubated for 30 min at room temperature. Fifty microliters stop reagent was then added to each well and absorbance was measured at 492 nm on a Multiskan Ascent Plate Reader (Thermo Fisher; Waltham, MA). LDH release was assessed by dividing individual absorbance values by the average Vehicle LDH release and graphed as fold change over Vehicle.

### Transfection with targeted siRNA

Fluorescein conjugated scramble siRNA (Santa Cruz Biotechnology; Dallas, TX), unconjugated scramble siRNA (Qiagen, Valencia, CA), or Tricellulin siRNA (Santa Cruz Biotechnology) were mixed 1:1 v/v with Lipofectamine 2000 (Invitrogen; Carlsbad, CA) in Opti-MEM medium (Life Technologies) to a final concentration of 400 pM and incubated for 30 min at room temperature protected from light. 2.5 × 10^6^ 16HBE14o- human bronchial epithelial cells were suspended in the resulting siRNA/Lipofectamine solution and incubated at 37 °C for 30 min. 5 × 10^5^ cells per well were seeded onto collagen coated Transwell inserts (Corning) and incubated at 37 °C. Six hours post transfection, equal volume of 16HBE14o- medium supplemented with 20% fetal bovine serum was added to the transfection solution in the apical chamber. Medium in the basolateral chamber was aspirated and replaced with 16HBE14o- medium supplemented with 10% fetal bovine serum. Medium was changed in both chambers to fresh 16HBE14o- medium supplemented with 10% fetal bovine serum 24 h post transfection.

### Western blot of tight junction proteins

16HBE14o- human bronchial epithelial cells or mouse lung tissue were homogenized and lysed in RIPA buffer (50 mM Tris-HCl, pH 8.0, 150 mM NaCl, 1% Triton X-100, 0.5% sodium deoxycholate) supplemented with protease and phosphatase inhibitor cocktail on ice and frozen at − 80 °C. Samples were thawed and centrifuged at 15,000 rpm for 15 min at 4 °C. Protein concentration was determined by Bradford assay and equal protein concentrations of lysate were run on a 5% stacking/10% separating SDS-PAGE gel. Proteins were transferred to PVDF membranes and blotted with 1:1000 rabbit anti-Tricellulin (Invitrogen 48–8400) or 1:50,000 mouse anti-GAPDH (Abcam ab8245) overnight at 4 °C followed by 1:10,000 horseradish peroxidase linked donkey anti-rabbit IgG (GE Healthcare NA934V) or horseradish peroxidase linked sheep anti-mouse IgG (GE Healthcare NA931V) for 1 h at room temperature. Signals were developed with Clarity western ECL substrate (Bio-Rad) and recorded with BioBlot BXR film (Laboratory Product Sales; Rochester, NY) or using Quality One software (Bio-Rad) with a Chemi Doc XRS scanner (Bio-Rad). Densitometry was assessed using ImageJ software.

### RT-qPCR of mouse lung tissue

Mouse lung tissue was snap frozen in liquid nitrogen and homogenized with Trizol reagent (Invitrogen). RNA phase was separated using chloroform extraction and RNA was collected using an E.Z.N.A. total RNA kit (Omega Biotek; Norcross, GA) according to manufacturer’s instruction. RNA was eluted in DEPC-treated water and RNA purity and concentration were measured using a NanoDrop spectrophotometer (Thermo Fisher). cDNA was reverse transcribed (RT) using an iScript cDNA synthesis kit (Bio-Rad) according to manufacturer’s protocol. Resulting cDNA template was added to wells containing 12.5 μl iQ SYBR Green Supermix (Bio-Rad) and 2 μl sense and antisense primers at a final concentration of 400 nM per primer (Tricellulin forward 5′ AATGACTCCTGAGCTGTTGAGTGG 3′, reverse 5′ TCCGCAGACAGCTCTTTGTACTCT 3′ or GAPDH forward 5′ CTTTGTCAAGCTCATTTCCTGG 3′, reverse 5′ TCTTGCTCAGTGTCCTTGC 3′). Wells were brought to a final volume of 25 μl using DEPC-treated water. qRT-PCR was run on a C1000 Touch Thermal Cycler (Bio-Rad) with the following protocol: An initial step at 95 °C for 3 min was followed by 40-cycle sequence of denaturation (30 s at 95 °C), annealing (30 s at 55 °C) and elongation (30 s at 72 °C). Data were evaluated by the 2^-ΔΔCT^ method [65].

### Generation of diesel aerosol

SRM 2975 DEP were suspended in water at 0.2 mg/mL with 5 μl/L TWEEN-80 (Millipore Sigma; St. Louis, MO). The resulting solution was mixed and sonicated with a probe sonicator (Sonics & Materials Inc.; Newtown, CT) at 750 watts, 20 kHz frequency, for 3 10 s bursts. Fifteen milliliters of this solution was put into an ultrasonic nebulizer (Model Ultrasonic2000, Nouvag Dental and Medical Equipment; Goldach, Switzerland) to generate the diesel mist. Clean dry dilution air (500–1000 ml/min) was passed through the nebulizer at a frequency of 2.4 MHz to produce the mist, which was then passed through a heated drying tube and cold trap to remove water. The resulting dry aerosol was mixed with diluting air and entrained into a 30 L stainless steel-reinforced Lexan exposure chamber at a rate of 25–30 L/min. Variation of the dilution air passing through the nebulizer controlled the concentration of aerosol that was delivered to the chamber. A peristaltic pump (Masterflex, Cole Palmer Inc.; Mount Vernon, IL) was used to maintain the diesel solution level in the nebulizer for the duration of the two-hour exposure session. Real-time exposure chamber particle number concentration was measured using a condensation particle counter (CPC, Model 3022A TSI Inc.; Shoreview, MN). The aerosol particle size distribution was assessed once per exposure using an electrostatic classifier (SMPS Model 3071, TSI Inc.; Shoreview, MN). Filter samples were periodically collected to determine gravimetric exposure concentration by mass. The goal was a nominal mass concentration value of 0.2 μg/L (200 μg/m^3^).

### Neonatal mouse whole body inhalation exposures

Adult male and female BALB/c mice were obtained from Jackson Laboratory and paired for breeding. After pairing, females were observed daily until vaginal plugs were noted. Upon identification of vaginal plugs, females were weighed, and male mice were removed. Fourteen days later, females were weighed to confirm pregnancy. Positive cages were observed daily until pups were noted. Starting between post-natal day (PND) 3 and 5, resulting male and female neonatal mouse pups were placed in individual wire mesh cages and placed inside a 30 L stainless steel-reinforced Lexan exposure chamber. Mice were exposed to aerosolized SRM 2975 or medical grade filtered air for 2 h per day for five consecutive days. Cages were rotated daily within the chamber to ensure even exposure throughout the exposure regimen. Two weeks after the final exposure, mice were euthanized, and lung tissue was collected for RNA and protein isolation. All mice were housed in an AAALAC, internationally accredited, specific pathogen-free vivarium facility with ad libitum access to rodent chow and water (12-h light/dark cycle). All procedures were reviewed and approved by the University of Rochester Committee on Animal Research.

### In vivo dosimetry estimation

In vivo particle dosimetry deposited dose was determined using the measured aerosol characteristics, estimated deposition fractions, and adjustments for neonatal mouse lung surface area. Using the Multi-Path Particle Dosimetry Model v 3.04 (MPPD v 3.04) [33]. Deposition fractions were estimated using the Multi-Path Particle Dosimetry Model [33] (MPPD v 3.04), the measured aerosol CMD and GSD, determined for the adult mouse using and allometrically-adjusted values for adult mouse tidal volume and respiratory rate [66]. Body weights for adult male and female and BALB/c mice were obtained using growth charts available from the Jackson Laboratory and averaged for use in allometric equations. The inhaled deposited dose was calculated using the airborne mass concentration, allometrically-adjusted lung physiology parametersminute ventilation [66], the sum of tracheobronchial and alveolar deposition fracitons, and assuming that no clearance occurred over the total 10 h of exposure. In order to account for differences in the adult and neonatal lung, we calculated the deposited dose over the entire surface area of the respiratory tract (adult mouse lung, ~ 500 cm^2^; PND 5 mouse lung, ~ 50 cm^2^ [34];).

### Statistical analysis

All values are expressed as mean ± standard deviation (SD). Exposure-related differences in measured outcomes were evaluated via Student’s t-tests, Unequal variances t-test, or one-way analysis of variance (ANOVA) followed by Tukey’s HSD group comparisons using GraphPad Prism software as indicated in the Figure Legends. Differences were considered to be statistically significant when *p* was less than 0.05.

## Supplementary information

**Additional file 1: Supplemental Figure 1.** TEER Values Normalized to Vehicle Treatment. TEER measurements of cells exposed to indicated concentrations of DEP at time 0, 6, and 8.5 h after application of DEP expressed as percent of average Vehicle TEER. One-Way ANOVA with Tukey’s HSD, ****P < 0.0001 Vehicle vs 25 μg/cm^2^ DEP, ##*P* < 0.01, ####*P* < 0.0001 Vehicle vs 50 μg/cm^2^ DEP, &*P* < 0.05, &&&& < 0.0001 25 μg/cm^2^ vs 50 μg/cm^2^ DEP. Data from three independent experiments, *N* = 5–6 replicates per treatment per time point per experiment.

**Additional file 2: Supplemental Figure 2.** PND 4–7 mice exhibit similar reductions to Tricellulin protein following aerosolized DEP exposure. A separate cohort of neonatal mice, exposed to aerosolized DEP starting between post-natal day 4–7, exhibit a similar reduction in Tricellulin protein in the lung 2 weeks post final exposure as measured by Western blot. Student’s t-test, *P < 0.05, *N* = 7 per treatment.

## Data Availability

The datasets used and/or analyzed during the current study are available from the corresponding author on reasonable request.

## Abbreviations

DEP: Diesel particulate matter/Diesel exhaust particles
TEER: Transepithelial electrical resistance
PM: Airborne particulate matter
AJCs: Apical junctional complexes
TJs: Tight junctions
TAMPs: Tight junction-associated MARVEL proteins
PND: Post-natal day
FA: Filtered air
MARVEL: MAL and related proteins for vesicle trafficking and membrane link
ECL2: Extracellular loop 2
AECs: Airway epithelial cells
ROS: Reactive oxygen species
MAPK: Mitogen activated protein kinases (MAPKs)
JNK: c-Jun N-terminal Kinase
ERK: Extracellular signal-regulated protein kinases
BSA: Bovine serum albumin
RT: Reverse transcribed
SD: Standard deviation
ANOVA: One-way analysis of variance
