# Supplementary figures and images for: Diesel exhaust particle exposure reduces expression of the epithelial tight junction protein Tricellulin
Source: Part Fibre Toxicol. 2020 Oct 15;17:52. doi: 10.1186/s12989-020-00383-x (PMC7560077; doi:10.1186/s12989-020-00383-x)

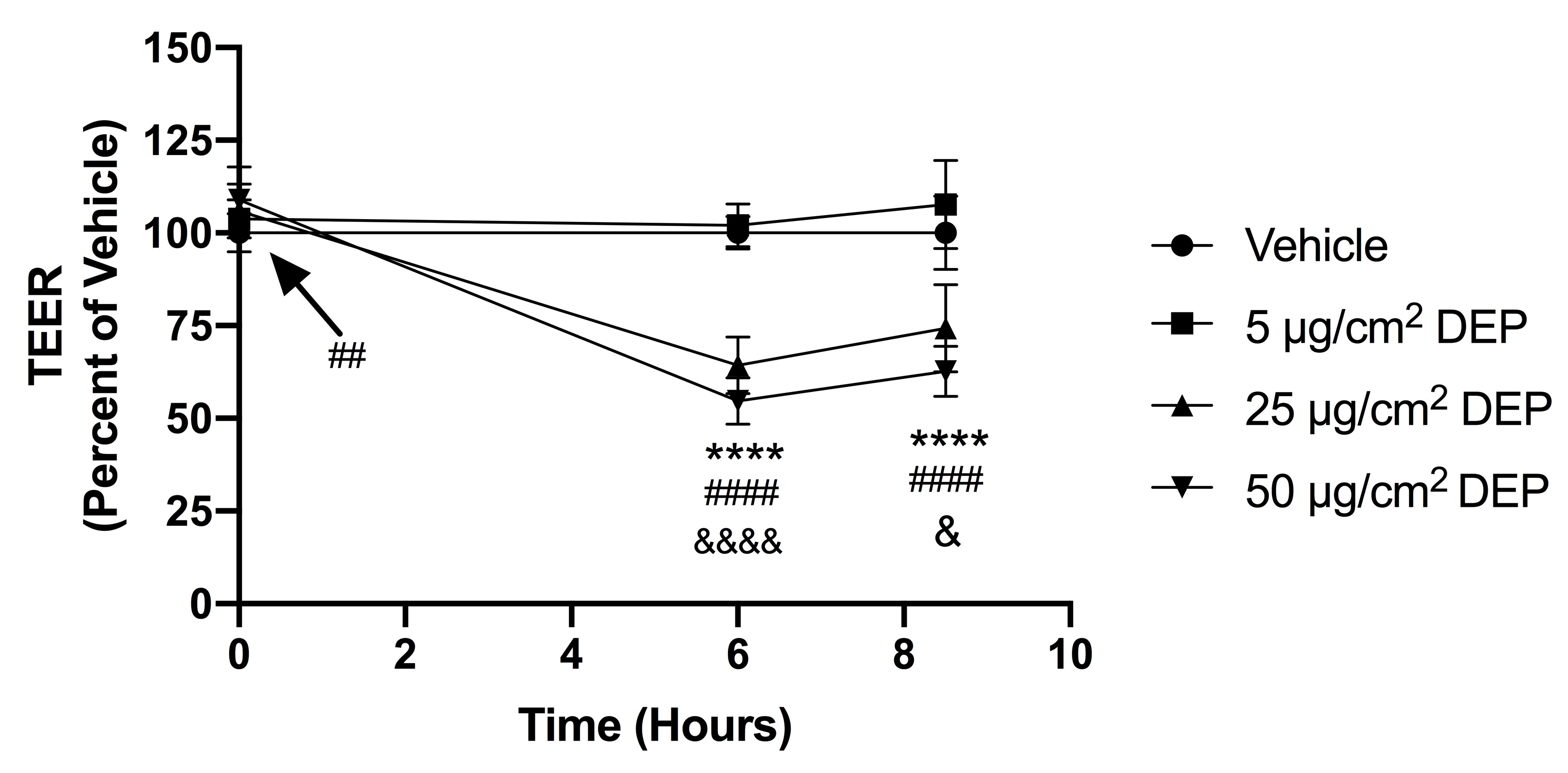

Supplement: Supplementary file 1 — Additional file 1: Supplemental Figure 1. TEER Values Normalized to Vehicle Treatment. TEER measurements of cells exposed to indicated concentrations of DEP at time 0, 6, and 8.5 h after application of DEP expressed as percent of average Vehicle TEER. One-Way ANOVA with Tukey’s HSD, ****P < 0.0001 Vehicle vs 25 μg/cm2 DEP, ##P < 0.01, ####P < 0.0001 Vehicle vs 50 μg/cm2 DEP, &P < 0.05, &&&& < 0.0001 25 μg/cm2 vs 50 μg/cm2 DEP. Data from three independent experiments, N = 5–6 replicates per treatment per time point per experiment. [file 12989_2020_383_MOESM1_ESM.jpg]

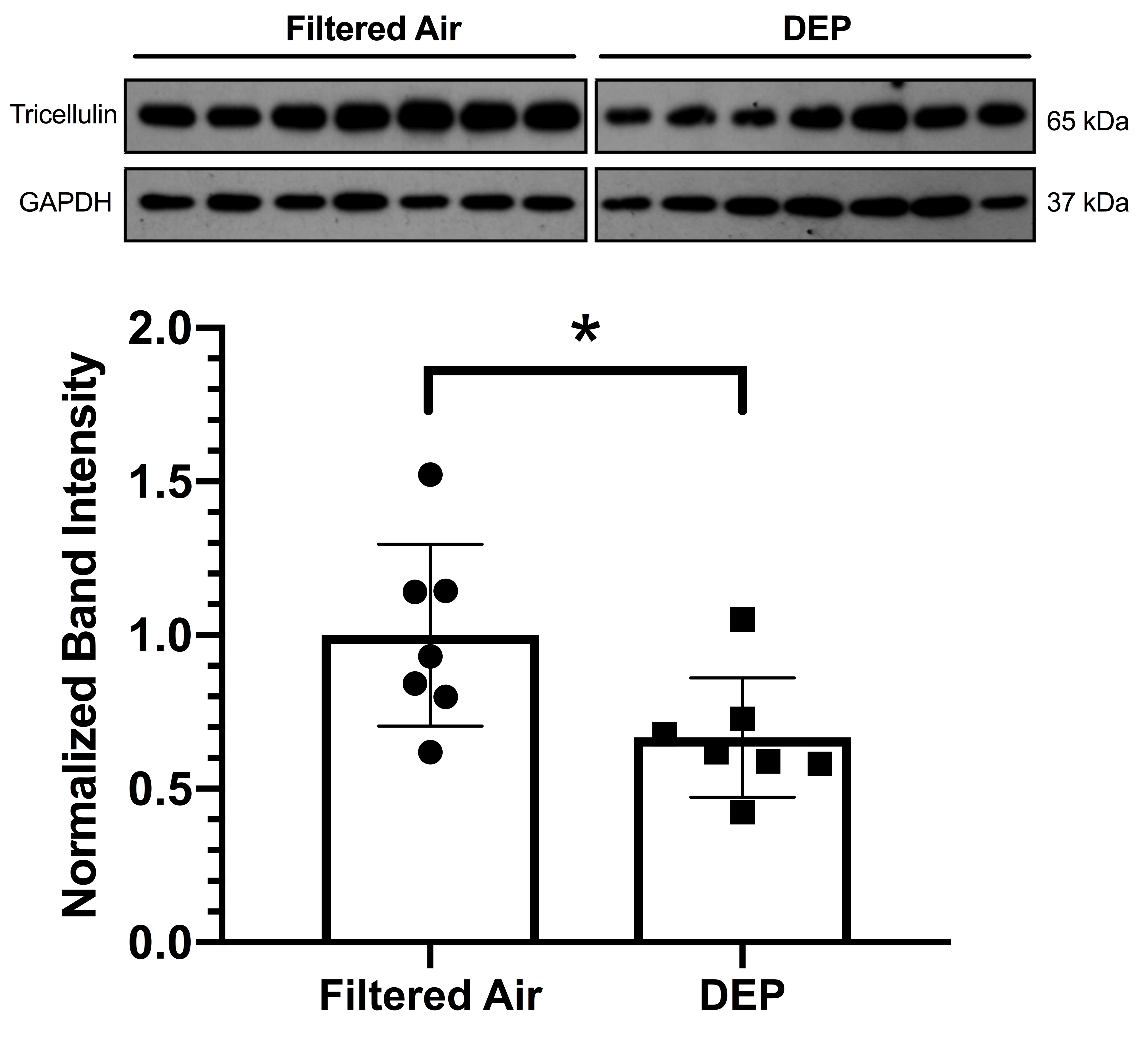

Supplement: Supplementary file 2 — Additional file 2: Supplemental Figure 2. PND 4–7 mice exhibit similar reductions to Tricellulin protein following aerosolized DEP exposure. A separate cohort of neonatal mice, exposed to aerosolized DEP starting between post-natal day 4–7, exhibit a similar reduction in Tricellulin protein in the lung 2 weeks post final exposure as measured by Western blot. Student’s t-test, *P < 0.05, N = 7 per treatment. [file 12989_2020_383_MOESM2_ESM.jpg]
